# Validation of a new measure of availability and accommodation of health care that is valid for rural and urban contexts

**DOI:** 10.1111/hex.12461

**Published:** 2016-05-18

**Authors:** Jeannie L. Haggerty, Jean‐Frédéric Levesque

**Affiliations:** ^1^ Department of Family MedicineMcGill UniversityMontrealQCCanada; ^2^ St. Mary's Hospital Research CentreMontrealQCCanada; ^3^ Centre for Primary Health Care and EquityUniversity of New South WalesSydneyNSWAustralia; ^4^ Bureau of Health InformationChatswoodNSWAustralia

**Keywords:** health services accessibility, outcome and process assessment, primary health care, questionnaire, validation studies

## Abstract

**Context:**

Patients are the most valid source for evaluating the accessibility of services, but a previous study observed differential psychometric performance of instruments in rural and urban respondents.

**Objective:**

To validate a measure of organizational accessibility free of differential rural–urban performance that predicts consequences of difficult access for patient‐initiated care.

**Design:**

Sequential qualitative–quantitative study. Qualitative findings used to adapt or develop evaluative and reporting items. Quantitative validation study.

**Setting:**

Primary data by telephone from 750 urban, rural and remote respondents in Quebec, Canada; follow‐up mailed questionnaire to a subset of 316.

**Main measures and analyses:**

Items were developed for barriers along the care trajectory. We used common factor and confirmatory factor analysis to identify constructs and compare models. We used item response theory analysis to test for differential rural–urban performance; examine individual item performance; adjust response options; and exclude redundant or non‐discriminatory items. We used logistic regression to examine predictive validity of the subscale on access difficulty (outcome).

**Results:**

Initial factor resolution suggested geographic and organizational dimensions, plus consequences of access difficulty. After second administration, organizational accommodation and geographic indicators were integrated into a 6‐item subscale of Effective Availability and Accommodation, which demonstrates good variability and internal consistency (α = 0.84) and no differential functioning by geographic area. Each unit increase predicts decreased likelihood of consequences of access difficulties (unmet need and problem aggravation).

**Conclusion:**

The new subscale is a practical, valid and reliable measure for patients to evaluate first‐contact health services accessibility, yielding valid comparisons between urban and rural contexts.

## Introduction

Accessibility is an attribute of health services. Services are accessible if their characteristics – location, organization, price, acceptability – fit with patients' ability to seek and obtain care.^1^, ^2^, ^3^, ^4^, ^5^ Although the health system or providers can validly and accurately report on location and how services are organized to meet the presumed needs of patients, the extent to which the services fit with the patients' actual abilities is most validly assessed from the patient perspective.

Variations persist in the definitions and conceptualizations of access,^3^, ^4^, ^6^, ^7^, ^8^ and these will have an impact on measure development. In this study, we are interested in first‐contact accessibility, including care seeking initiated by the patient. First‐contact accessibility is defined operationally as ‘the ease with which a person can obtain needed care (including advice and support) from the practitioner of choice within a time frame appropriate to the urgency of the problem’.^9^ The experience of first‐contact accessibility begins with decision to seek care and ends when needed services are obtained.

Patient‐initiated care seeking involves three subdimensions of accessibility that were defined in recent literature synthesis of that led to a conceptual framework of patient‐centred accessibility.^8^ Approachability is the extent to which services are known as geographically or socially reachable patients; this includes communicating what is offered to a wide range of potential patients. Availability refers to the physical presence or location of a service that allows it to be reached easily (sometimes referred to as geographic accessibility). Accommodation refers to how services are organized to permit patients with a wide range of abilities to obtain their care, including opening hours, communications and interactions with staff.

Ensuring timely accessibility is an issue in all health systems, but especially in the Canadian context where this has been problematic over the last years. It is critical to have accurate and comparable accessibility measures to track the performance of the health system, and particularly of primary health care, which has the responsibility of ensuring first‐contact access.

A counterintuitive finding in a 2002 survey of primary care clinics across the province of Quebec, Canada, motivated this study. We found that rural patients evaluated their ability to be seen rapidly and their clinic's organizational accommodation more positively than did urban patients – despite longer distances, fewer local options, shorter office hours and observed longer wait times for routine care.^10^, ^11^ Other studies have also found that rural residents provide more positive assessments of accessibility than do their urban counterparts.^12^, ^13^ As ensuring equitable access to services across geographic contexts is an enduring concern for health planners, we set out to determine whether the finding was a measurement artefact. Indeed, we found evidence of differential psychometric performance by geographic context in the validated measures of accessibility that we used.^14^ Specifically, most items in the First‐Contact Access subscale of the Primary Care Assessment Tool (PCAT)^15^ and the Organizational Accessibility subscale of the Primary Care Assessment Survey (PCAS)^14^, ^16^ demonstrate higher discriminability and reliability in urban than in rural respondents. When differentially performing items were removed, the previously observed difference between rural and urban respondents was either substantially diminished or was reversed. Such differential item functioning compromises the capacity to compare health‐care access validly and reliably across urban, rural and remote areas.

We conducted a mixed‐method sequential qualitative–quantitative study where our objective was to refine and develop accessibility measures that would be equally reliable and valid in both rural and urban areas. In the first and qualitative phase, we explored through 11 focus groups the similarities and differences between metropolitan, rural and remote settings in the first‐contact care‐seeking trajectory. We found that rural residents invested more efforts than did urban residents in exploring care alternatives before travelling to their preferred option and that their regular providers were more likely to accommodate to their urgent needs.^17^ However, the consequences of access difficulties were similar across settings: having to restart the care‐seeking process; abandonment of care seeking; resorting to the hospital emergency room; and/or aggravation of the health problem.

In this article, we report on the quantitative phase of the mixed‐method study. The objective was to develop a new accessibility measure that is sensitive to rural care‐seeking trajectories that emerged in our qualitative phase and to determine whether the measure is equally valid and reliable in rural and urban respondents.

## Methods

We conducted this sequential mixed‐method study between 2004 and 2010 in Quebec, Canada. Our study population was predominantly French speaking and was selected to represent urban, rural and remote populations. The study received research approval from the research ethics committees of the University of Montreal Research Center and the Charles Lemoyne Hospital Research Center.

### Development and adaptation of items

The codes from the focus groups analysis (qualitative phase) were stated as quantifiable elements facilitating or impeding timely access or as consequences (summarized in the introduction and detailed elsewhere).^17^ We identified nine validated subscales for first‐contact accessibility in the literature, and we mapped the items to the codes that were most frequently invoked. Mapping revealed several rural‐specific barriers or facilitators that were not covered in any instruments, such as care seeking by telephone, organizational flexibility to accommodate individuals and some space/time barriers. For every barrier or facilitator, we suggested indicators. Where possible, we adapted items from the validated instruments on the premise that they had demonstrated adequate metric properties. We developed new items for barriers or facilitators not covered in any instruments. We developed items simultaneously in French and English to achieve semantic equivalence.

### Measurement approach

We used a mix of evaluative and reporting indicators to elicit positive and negative experiences of accessibility. Guided by our operational definition of first‐contact accessibility, we used an ease‐of‐access 5‐point Likert's scale (1 = not at all easy to 5 = very easy) for evaluative items. For reporting items, we elicited frequencies (1 = never to 5 = almost always) and self‐reported estimates of occurrence to triangulate the frequency scaling.

Typically, accessibility surveys exclude respondents without recent service use, but as non‐use may reflect access problems, we adapted our instrument to include them by eliciting expected experience based on their most recent experience. The instrument framed the items by asking respondents to envision getting sick and needing care or advice. Similarly, for respondents without a regular source of care or physician, we targeted the experience towards their most frequently or recently consulted source of care.

We conducted cognitive testing on convenience samples of French and English speakers with no more than secondary school education (initially in person, then by telephone) to ensure respondents understood the statements as intended and that response options were relevant to their experience. We excluded several items at this stage: organizational accommodation processes that patients did not observe or experience directly, rare events or statements for which French and English equivalents could not be found.

The initial instrument contained 37 items organized along the typical care‐seeking trajectory. We purposefully added redundant items, including 13 from validated instruments, so that we could select the best‐performing ones. After psychometric analysis, we modified and shortened the instrument, leaving 22 items measuring geographic accessibility, organizational accommodation and consequences of access difficulty. Questions were included on health‐care use, general health status, and social and demographic characteristics.

### Instrument administration

For initial validation, we administered the questionnaire using computer‐assisted telephone interviewing to 750 respondents selected by random‐digit dialling: 250 each from an urban, a rural agricultural and a remote area (more than 3 h from a tertiary care centre). The programme adapted questions depending on whether respondents had used services within the past 12 months or had a regular source of care or physician. We asked permission to contact respondents for a second administration. The resulting subsample was mailed a self‐administered version of a refined and shortened questionnaire.

### Analysis

Items with >4% missing values and lack of variance or floor/ceiling effects (whether globally or by geographic, educational or service use categories) were excluded or targeted for improvement if the domain was critical. We examined Pearson and Spearman correlations among all variables and conducted exploratory common factor analysis to assess whether and how well items loaded on expected factors.

To maximize sample size for psychometric testing in initial exploratory factor analysis, we imputed missing values using the conservative Monte Carlo method,^18^ excluding six respondents with more than 10 missing values. We conducted factor analysis in the subset of 655 recent service users then tested for differences taking into account non‐users' expected experiences. Within each factor, we examined individual item performance using item‐total correlations and nonparametric and parametric item response theory (IRT) analysis.^19^, ^20^ This allowed us to compare the discriminability and information yield of new items with those of validated items and to determine which response option formats performed optimally. We eliminated poorly functioning or redundant items. We favoured newly developed items over validated items where possible.

For the self‐administered questionnaire, we confirmed factor structures using structural equation modelling,^21^ eliminating variables when goodness of fit of the factor resolutions improved by their exclusion from the model. Using parametric and nonparametric IRT analyses, we further eliminated items that did not have good discriminatory capacity or make a unique contribution to the information yield for the construct, or where the response options did not function as intended. We tested for differential item functioning by geographic context using parametric IRT. Finally, for predictive validity we used logistic regression modelling with SAS9^22^ to examine whether the odds of indicators of difficulty of access (dependent variable) met ordinal and interval assumptions for each unit change in item and subscale scores (independent variable).

## Results

Of the 750 persons who participated in the telephone administration of the initial questionnaire, 492 (66%) agreed to be re‐contacted. Of these, 93 were unreachable; of the remaining eligible persons reached, 31 refused and 52 did not return the questionnaire (response rate 79.2%, *n* = 316/399). Table 1 presents the socio‐demographic, health and health‐care use characteristics of both samples. Those not responding to the second administration were less likely to have a personal physician and had slightly less education but did not differ in health‐care use or geographic distribution.

**Table 1:** Socio‐demographic, health and health‐care utilization characteristics of validation samples

Characteristics	Initial sample (*n* = 750)	Repeat sample		Test for difference by response (repeat sample)
		Respondents (*n* = 316)	Non‐respondents (*n* = 434)	
Sociodemographic				
Mean age (SD)	50.9 years (15.5)	51.4 years (14.4)	50.6 years (16.2)	*t* = −0.72; *P* = 0.47
Per cent female	69.6% (522)	71.8% (227)	68.0% (295)	χ^2^ = 1.29; 1 d.f.; *P* = 0.26
Geographic context				
Urban	33.3% (250)	31.7% (100)	34.6% (150)	χ^2^ = 0.83; 2 d.f.; *P* = 0.66
Rural	33.3% (250)	34.8% (110)	32.3% (140)	
Remote	33.3% (250)	33.5% (106)	33.2% (144)	
Per cent with at least high school education	22.7% (170)	24.1% (76)	21.7% (94)	χ^2^ = 7.87; 3 d.f.; *P* = 0.049
Health				
Per cent rating health as very good or excellent	53% (397)	53.9% (170)	52.3% (227)	χ^2^ = 3.47; 4 d.f.; *P* = 0.48
Per cent with a chronic physical condition	36.7% (274)	39.8% (125)	34.5% (149)	χ^2^ = 2.21; 1 d.f.; *P* = 0.14
Per cent with important limitation for daily activities	23.7% (178)	26.3 % (83)	21.9% (95)	χ^2^ = 1.93; 1 d.f.; *P* = 0.16
Health‐care utilization				
Per cent with usual source of care	92.1% (691)	91.5% (289)	92.6% (402)	χ^2^ = 0.35; 1 d.f.; *P* = 0.56
Per cent with personal physician	81.2% (609)	84.5% (267)	78.8% (342)	χ^2^ = 3.88; 1 d.f.; *P* = 0.048
Per cent using health‐care services in past year	87.3% (655)	89.2% (282)	85.9% (373)	χ^2^ = 1.80; 1 d.f.; *P* = 0.18

Only 95 (12.7%) of initial respondents had not used services in the past 12 months; none had missing values for expected experience of care items. However, among the 655 (87.3%) who reported recent experience of care, missing values were common, as not all users experienced every stage of a typical care trajectory in their last encounter. This led to our decision to elicit expected experience with organizational accommodation so that questions could be answered by all respondents.

**Figure 1:**
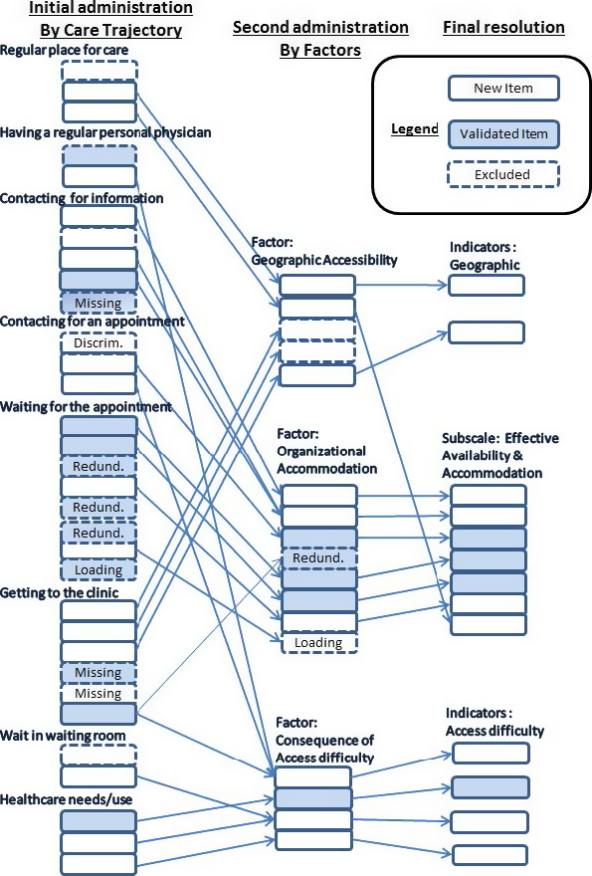
Overview of instrument evolution: first administration (items grouped by care trajectory); second administration (items grouped by factors); final instrument. Arrows show movement of items between versions.

Figure 1 displays the initial instrument structure (items organized by stages of care trajectory), the instrument at second administration (items grouped by factors emerging from initial analysis) and the final instrument (Effective Accessibility and Accommodation subscales). Initial exploratory analyses found three groupings of factors: geographic (space/time considerations), organizational accommodation and consequences of difficulty of access. Where validated and new items were correlated (*r* >0.50) and provided similar information, we kept the new items; if a validated item performed better, we adapted the statement to fit our response options. Here, we present the detailed results of the psychometric analyses of the second administration and discuss the Effective Accessibility and Accommodation subscale.

### Geographic availability

Items loading on the factor presumed to measure geographic availability elicited information on clinic location, ease of travel to clinic for urgent or routine care and local availability of alternatives. The number and type of response options varied in the initial administration. In the factor analysis, loadings were modest and internal consistency low (α = 0.64). These metrics were expected to improve after adapting responses to the 5‐point Likert's scale options offered in the second administration. The item statements, response options and descriptive statistics are shown in Table 2.

**Table 2:** Item statement, response distribution, and factor loadings for proposed geographic availability subscale, second administration (*n* = 316)

	Median, mode (IQR)^a^	Mean (SD)	Factor loading	Remark
**Geographic availability**				
1. Is the clinic close to your home? (1 = very far, 5 = very close)	4, 4 (1)	3.79 (0.96)	0.78	*r* = 0.78 correlation between both items
2. How long does it take you to get to your clinic? (1 = more than one hour, 5 = less than 5 min)	4,4 (1)	3.72 (0.97)	0.74	
3. For your *routine or non‐urgent* health‐care needs, how easy is it to travel to your clinic? (1 = not at all easy, 5 = very easy)	4, 5 (2)	3.74 (1.21)	0.54	*r* = 0.66 correlation, but low correlation with proximity items (*r* ~ 0.29)
4. When you need *immediate* care, how easy is it to get to your clinic? (1 = not at all easy, 5 = very easy)	4, 5 (3)	3.40 (1.38)	0.57	
5. What phrase best describes the number of healthcare clinics present in your neighbourhood? (1 = none for miles, 5 = many clinics nearby)	3, 3 (1)	3.30 (0.89)	0.24	Best descriptor of context
Subscale score (Items 1–5)	3.60, 3.20 (1.0)	3.59 (0.73)	*(*α *= 0.68)*	

a IQR = Interquartile range, 75th–25th percentile, estimate of spread for ordinal variable. Although not appropriate for ordinal values, this provides a typical measure of central tendency and spread. Scored from 1 (poorest expected accessibility) to 5 (best expected accessibilty). Scored initially as sum of ‘1 = yes’ responses (range 0– 4), transformed into 1–5 scale.

Words in italics were formatted to give special emphasis in the presentation of the item.

Common factor analysis of the second administration still did not produce factor loadings suggestive of a single coherent structure (Table 2), and internal consistency remained low (α = 0.68). The first two items on clinic proximity were strongly correlated (*r* = 0.78), as were the ease‐of‐travel items (*r* = 0.66), but proximity items correlated only modestly with ease of travel (*r* ~ 0.29). Correlations did not improve when controlled for physical limitations, vehicle ownership or geographic context, suggesting these indicators are associated with slightly different underlying constructs.

The item eliciting perceived local availability of clinics correlated weakly with the other items (*r* = 0.09–0.22), had a low item‐total correlation (0.21) and its removal improved the subscale's internal consistency (α = 0.73). However, this item discriminated well between urban, rural and remote contexts (MH χ^2^ = 75, phi = 0.56, Somer's *D* = 0.48), and the respondents' responses corresponded well to an independent measure of density of primary care and hospital resources in the geographic areas. This suggests that the item is valid and discriminatory indicator but is not internally consistent with the rest of the items in the construct.

Item response theory analysis suggested a 3‐point scoring for each item, and summing the item scores improved internal consistency and interpretability. Overall, however, the analyses did not strongly support a single underlying construct for these five items. Logistic regression modelling of access consequences on the subscale and on individual items (reported below) led us to conclude we had good indicators of geographic accessibility, but not a robust, reliable and valid subscale *per se*.

### Organizational accommodation

The initial factor resolution for organizational accommodation included indicators from most stages of the care trajectory (Fig. 1). The adequate internal consistency (α = 0.73) was expected to improve on the second administration. An item expected to indicate geographic availability (possibility to avoid travel by getting telephone medical advice) loaded highly with other organizational accommodation items, as did the ease‐of‐travel items, presented above, for geographic availability. We modified some items. For example, we simplified a new item eliciting ease of getting medical information by telephone because, despite having lower discriminability than two similar validated items, it had better information yield for positive accessibility and higher discriminability in rural than in urban populations.

Table 3 shows the eight questions used to elicit organizational accommodation in the second administration, along with descriptive statistics, factor loading results and final decision. The full range of response options was endorsed for every item. Although frequency distributions were skewed towards positive assessments, measures of central tendency varied, suggesting varying degrees of difficulty that enhance the scale's information yield. All but one item (item 8) loaded well on a single factor, but the high resulting internal consistency (Cronbach's α = 0.88) suggested possible redundancy, so we used IRT analysis to drop two items. Of two items (3 and 4) asking about getting medical advice by telephone, we preferred item 3 because it yielded more information in the most positive zone of accommodation. Item 8, clinic structures that facilitate rapid access, was designed as an additional indicator of organizational accommodation, but it loaded weakly on the underlying factor. It was dropped because IRT analysis demonstrated that ‘yes’ and ‘no’ response options were very discriminatory for positive and negative organizational accommodation, respectively, but ‘don't know’ was endorsed across the entire range of organizational accommodation, compromising item information yield and subscale reliability. Finally, to represent all stages of the care trajectory, we added an indicator of geographic availability – ease of travel to the clinic – that loaded well (0.62) with other items in the Organizational Accommodation subscale.

**Table 3:** Item statement, response distribution, and factor loadings for proposed organizational accommodation subscale, second administration (*n* = 316)

*Imagine that you are sick and you need care or medical advice…* (1 = not at all easy, 5 = very easy; exceptions in response options indicated)	Median, mode (IQR)^a^	Mean (SD)	Factor loading	Remark
1. Based on your experience, how easy would it be for you to get health care or advice from your clinic?	4, 4 (1)	3.48 (1.20)	0.80	
2. If you were sick, how easy would it be for you to reach the clinic over the phone?	4, 5 (2)	3.66 (1.34)	0.71	
3. How easy would it be for you to get medical advice from the clinic over the phone?	3, 4 (2)	2.94 (1.45)	0.79	
4. If you have a question or need medical advice, how easy would it be for you to reach your doctor and to talk to him over the phone?	2, 1 (3)	2.58 (1.40)	0.72	Dropped, redundant with #3
5. What is the usual wait for an appointment with your doctor? (categories discretionary, not used in subscale score)	4, 3 (3)	3.65 (1.65)	–n/a	Not used in subscale score
6. How do you rate the usual wait for an appointment with your doctor? (1 = very poor, 5 = very good)	3, 3 (2)	3.05 (1.20)	0.66	
7. At your clinic, if you need to be seen quickly, how easy would it be to be seen sooner than the usual appointment time?	4, 4 (2)	3.27 (1.37)	0.74	
8. Does your clinic do the following things to help you to get care or medical advice rapidly? (1 = yes, 0 = no/don't know)	2, 2 (1)	2.46 (1.01)	0.46	Dropped, low information yield
a. Offers regular walk‐in services				
b. Provides medical advice by telephone				
c. Offers you a visit with another doctor				
d. Offers to see you between scheduled visits				
New From Geographic How easy is it usually to travel to your clinic? (1 = not at all easy, 5 = very easy)	4, 5 (2)	3.74 (1.21)	0.62	Added to final subscale (from Item 3, geographic)
Subscale score	3.50, 4.33 (1.57)	3.36 (1.01)	α* = 0.84*	

a IQR = interquartile range, 75th–25th percentile, estimate of spread for ordinal variable. Although not appropriate for ordinal values, this provides a typical measure of central tendency and spread. Scored from 1 (poorest expected accessibility) to 5 (best expected accessibilty). Scored initially as sum of ‘1 = yes’ responses (range 0–4), transformed into 1–5 scale.

Words in italics were formatted to give special emphasis in the presentation of the item.

The subscale score is the median value of the items. The median as the measure of central tendency respects the formal mathematical assumptions for ordinal categorical variables. For the validation sample, the median was 3.5, corresponding approximately to ‘less than moderately easy’. The subscale score demonstrated good variability, with scores ranging from 1.0 to 5.0. Finally and importantly, we found no differential item functioning by geographic context.

### Consequences of access difficulty

Our focus groups identified four consequences of access difficulty that occur across different geographic settings and at almost every stage of care seeking. The first and most frequent was nuisance – having to restart all or part of the process after encountering impediments. The others were more significant: unmet need (abandoning the care‐seeking process), emergency room use (bypassing primary care), and health problem aggravation because of delays in getting care. Eliciting the occurrence of these consequences and their attendant reasons (Table 4) provided a prevalence of access difficulties.

**Table 4:** Indicators of consequences of difficult access

In the last 12 months… (1 = Never, 2 = Sometimes, 3 = Often)	If sometimes or often, what was the reason? Check as many as apply (list of system reasons)		
*Nuisance* …did you have to make several attempts to get the health care you needed because of the difficulties you encountered?	No	Yes	
	□	□	Because your regular doctor was not available
	□	□	Because nobody was available to see you at your regular clinic
	□	□	Because you did not have a regular doctor or clinic
*Unmet Need* …was there ever a time you felt you needed health care but didn't get it?	□	□	Because it was too difficult to make an appointment
	□	□	Because the wait for an appointment was too long
	□	□	Because the wait in the waiting room was too long
*Emergency Room Use (For System Reasons)* …did you go to a hospital emergency room for health care?	□	□	Because the clinic was not open during hours you could attend
	□	□	Because the clinic was too far or it was difficult for you to get there
	□	□	Because you did not feel comfortable with the available doctor or nurse
*Problem Aggravation* …did a health problem ever become more serious because it took a long time to get health care?	□	□	Because you had an appointment but did not see the doctor yet
	Other:		––––––––––––––

Nuisance was the most common though minor consequence, reported as occurring ‘rarely’ or ‘sometimes’ by 43% and ‘often’ by 7% (Table 5). Emergency room use was reported by 35.7% (12.3% for reasons specific to the health system). Problem aggravation was least common. Of respondents reporting a major consequence, 21% experienced more than one. Unavailability of a personal physician was the most commonly cited reason for all consequences, followed by long wait for an appointment.

**Table 5:** Prevalence of access difficulties and their likelihood of occurring with each unit increase in measure of accessibility^a^

	Consequences of access difficulty			
	Nuisance	Emergency room use, system reasons only	Unmet need	Problem aggravation
Prevalence % (*n* = 316)	50.1% (158)	12.3% (38)	21.9% (67)	8.4% (26)
Odds ratios for geographic accessibility items (95% CI)				
Perceived clinic proximity	0.83 (0.63, 1.09)	0.91 (0.63, 1.32)	0.95 (0.71, 1.26)	1.10 (0.71, 1.68)
Ease of travel, routine	0.64 (0.52, 0.79)***	0.96 (0.74, 1.24)	0.53 (0.42, 0.67)***	0.96 (0.79, 1.30)
Availability of nearby alternatives	0.64 (0.46, 0.88)**	0.53 (0.35, 0.81)**	0.77 (0.56, 1.05)	0.71 (0.45, 1.11)
Odds ratios for effective availability and accommodation subscale (95% CI)				
Effective availability and accommodation subscale	0.33 (0.24, 0.46) ***	0.69 (0.49, 0.97)	0.39 (0.28, 0.53)***	0.43 (0.27, 0.68)***

a Separate logistic regression models showing odds ratio of consequence associated with each unit increase in accessibility, controlling for physical limitations and chronic illness; 5‐point scale.

*P <0.05; **P <0.01; ***P <0.001.

Table 4 shows the relationships between geographic accessibility items and access difficulties. Better perception of proximity to the regular clinic is associated with lower risk of nuisance, of unmet need, and of problem aggravation, with stronger effects in rural areas. Increased ease of travel to clinic for routine care is associated with reduced likelihood of both nuisance and unmet needs. Availability of nearby alternatives is associated with reduced likelihood of both nuisance and emergency room use. These effects are stronger in rural areas and for persons self‐identifying as poor. Ordinal logistic regression confirmed that the item score effects were ordinal, but also suggested effect thresholds (details available on request).

Each unit increase in the effective availability and accommodation scores decreased the likelihood of nuisance, of unmet needs, and of problem aggravation but not of emergency room use (Table 5). There was significant modification of the association between unmet needs and effective availability and accommodation by geographic setting: in remote and rural areas (no clinics nearby), even a small increase in effective availability and accommodation could reduce unmet needs to negligible amounts, whereas in urban areas (many clinics nearby) the effect was modest, although still statistically significant.

## Discussion

This study led to the development of an organizational accommodation measure, the Effective Availability and Accommodation subscale, which is free from differential functioning between urban and rural respondents. The indicators we proposed for a subscale measuring geographic accessibility did not hold as a single factor, but the component items function as useful indicators of different space/time barriers. The consequences of access difficulty can also be used as an independent measure to compare accessibility across geographic contexts and population groups.

This subscale will be of particular relevance to health service planners and evaluators and rural health services researchers. It is very important that accessibility measures be free of differential functioning by geographic context so that they can be used to compare health service accessibility between urban and rural areas. In a publicly funded system, health planners monitor a variety of access indicators to determine the placement and resourcing of health‐care facilities. Although this measure contains some items that are more discriminating in one context than another, the differences are not statistically significant, and overall, the subscale is free from bias.

Bias in the measures may account for some of the findings in other studies that rural residents provide more positive assessments of accessibility than do their urban counterparts,^12^, ^13^ but not all. In the qualitative component of our study, we found that primary care practices in rural and remote areas seemed more likely to accommodate to individuals' needs than did urban practices and that people could more easily mobilize their social networks to facilitate their care trajectory.^17^ Our subscale may be used as an alternative to the Organizational Accessibility subscale in the Primary Care Assessment Survey,^16^ from which it is heavily influenced, with the confidence that the new measures detect meaningful differences between geographic contexts and practices.

Consequences of difficulty of access can also be used as valid comparators between rural and urban areas. The type and frequency of consequences related to difficulty in accessing care are similar by context, with the exception of emergency room use for low‐acuity problems. In rural areas where care alternatives are constrained, emergency rooms are often used for urgent as well as emergency care.^23^ Emergency room use is widely used as a proxy measure of problems in accessing primary health care.^24^, ^25^ Consequently, our measure also elicits system‐induced use, which we believe may be a more accurate indicator of problematic accessibility of health systems. Unmet need is a widely used measure, and our study supports its sensitivity to organizational processes, geographic context and personal characteristics. Finally, health problem aggravation due to delay is a new indicator that is sensitive to effective availability and accommodation and to contextual and individual differences. Nuisance is not a major consequence, but given its frequency, low prevalence may indicate good accessibility.

### Effective Availability and Accommodation subscale

The Effective Availability and Accommodation subscale includes items from every stage in the care‐seeking process, including ease of travel to the clinic. It elicits experience of telephone access and organizational flexibility, which our focus groups identified as distinctive elements of the rural care‐seeking trajectory. The subscale is internally coherent, and higher scores are associated appropriately with lower likelihood of consequences of difficult access. The items do elicit perceived urgent need based on past experience, making them valid for different utilization patterns, although it would be prudent to analyse by recent use.

The subscale purports to capture the construct of availability and accommodation suggested by Levesque *et al*.^8^ We qualify availability as effective as per Frenk,^3^ who proposed that characteristics of available health services create resistance (obstacles) in the care‐seeking process and that effective availability is a function of the extent to which those points of resistance interact with users' ‘utilization power’ to overcome them (utilization power being analogous to consumers’ purchasing power to obtain commercial goods.) The item statements in our Effective Availability and Accommodation subscale represent common obstacles in the care‐seeking process; the response options reflect the ease with which individuals overcome them. The perceived difficulty of each obstacle varies by individuals' intrinsic utilization power, including perception of need, knowledge of the options for care (including self‐management), acceptability of the options based on prior experience, access to transportation, ability to pay and ability to obtain needed information. It is important to analyse the subscales and items (obstacles) by subgroups that may be particularly disadvantaged. For instance, we found that respondents in lower socio‐economic groups were more likely to experience the access consequences for each of the obstacles and overall; it was also clear in our focus groups that rural residents without personal vehicles experienced travel to the clinic as a major barrier to seeking care. The suggested scoring for the subscale (median of all item values) implies the obstacles are cumulative and of equal weight. This assumption remains to be tested in independent samples, preferably in different health‐care contexts. Ultimately, the subscale is designed to reflect on the extent to which service providers organize their health services in a way that allows a wide range of potential users – and not only those endowed with greater utilization power – to realize access in response to self‐perceived need.

Two widely used validated subscales were influential in our work. The First‐Contact Access subscale of the PCAT^16^ elicits the probability of being seen or getting information under different service availability scenarios. Its hypothetical framing inspired us to explore and compare assessments of direct and expected experience. The Organizational Accessibility subscale of the PCAS has excellent psychometric properties^16^, ^26^ but seems to reflect an urban care trajectory. We adapted the indicators to elicit perceived ease rather than satisfaction ratings, resulting in less skewed distributions that provide better discriminability on both urban and rural subgroups. Our modifications overcame the problem that these two subscales demonstrated of being more discriminating in urban than in rural populations.^14^

### Indicators of geographic availability

In the initial stages of our endeavour, we were not able to identify measures of geographic availability of health services from the patient perspective, and so we set out to develop such a measure. In our focus group study, transportation and travel emerged as obstacles in the care trajectory, but their importance in impeding care depended on many factors, including nature of the health problem (urgency, perceived severity); preference for a specific provider; availability of transportation; physical mobility; and opportunity or costs associated with care alternatives.^17^

Our proposed indicators of space/time dimensions of access did not load convincingly on a single underlying construct. The items we used appeared to measure slightly different, though related, constructs: proximity to usual clinic, travel time, ease of travel and local alternatives. The ease‐of‐travel item became part of the Effective Availability and Accommodation subscale. Proximity of the clinic and travel time are good descriptors of geographic accessibility, but only the most extreme values of distance from services predicted access difficulties. Local availability of clinics corresponds well to objective measures of health service density, making it a good indicator of geographic context. We were disappointed that we were unable to produce a robust measure of geographic access, although individual indicators were appropriately sensitive to geographic context and predicted access difficulties. We hope our work will contribute to future work to develop a reliable measure of geographic accessibility.

## Limitations

An important limitation of this study is the generalizability of its results. The measure was developed and validated in Quebec, Canada, and although we tried to express barriers and facilitators in general terms, their relative importance or validity may be specific to that specific context. In this publicly funded health system, patients can ostensibly choose providers, but actual choices are constrained by a shortage of family physicians accepting new patients. In a context of low supply and high demand, first‐contact services have little incentive to accommodate the needs of a wide range of patients. From a measurement perspective, this creates a variance in patient experience that enhances the reliability and predictive capacity of our measure. In different contexts where health services are either generally or not at all accommodating, reliability may be compromised. We did find, however, that the frequency distributions of experience were less skewed with our measure than with other validated measures used in this same context.

The items that compose the Effective Availability and Accommodation subscale should help evaluators or providers identify specific organizational dimensions of accessibility that are problematic and can be modified. We believe that the items and implied care trajectories are relevant for most industrialized health‐care contexts. We can imagine that items such as telephone access or ease of travel may not be sensitive indicators in rural contexts where telephone costs could be high or where online physician consultations are readily available. The subscale is predicated on having a regular place of care and on the assumption that accommodation is determined predominantly by organizational arrangements. In some contexts or populations where cost is the major barrier, the measure of accommodation will be of secondary importance compared to a measure of affordability. Costs of services may be health system dependent; elsewhere, we report on a subscale of health‐care affordability for Canada's publicly funded system.^27^ The validity and reliability of the indicators and subscale need to be tested in other contexts. In contrast, we believe that the indicators of consequences of difficult access will be robust across health systems, including middle‐income countries.

A potential limitation of our study is that our classification of urban and rural may not concord with other definitions. Indeed, ours is at variance with that of Statistics Canada,^28^ which identifies what we called rural towns as small urban agglomerations. However, participant from towns in this study self‐identified themselves as rural, both culturally and with respect to access to a full range of health services. We believe that recognizing different rural contexts – towns, rural agricultural villages and remote rural villages – is a strength of our study.

## Conclusion

We have refined and validated a measure of effective availability and accommodation. The measure is specific to first‐contact accessibility and is therefore more relevant to assessment of primary health care. The measure is equivalently reliable and valid for rural and urban contexts, so it can be used to compare health‐care accessibility by geographic context. We also propose a set of indicators that we believe can be used to compare access difficulties validly across geographic contexts. The measure was developed and validated in Quebec, Canada; it remains to be validated in other geographic and health system contexts.

## Funding

This study was supported by the Canadian Health Services Research Foundation (now Canadian Foundation for Healthcare Improvement), Charles‐LeMoyne Hospital Foundation and St. Mary's Hospital Foundation.

## Conflict of interest

Neither Jeannie Haggerty nor Jean‐Frédéric Levesque have any financial or non‐financial conflicts to declare.
